# NCAPG as a Novel Prognostic Biomarker in Glioma

**DOI:** 10.3389/fonc.2022.831438

**Published:** 2022-02-23

**Authors:** Xiulin Jiang, Yulin Shi, Xi Chen, Haitao Xu, Bohu Liu, Fan Zhou, Xiaobin Huang, William C. Cho, Lihua Li, Jun Pu

**Affiliations:** ^1^Department of Neurosurgery, The Second Affiliated Hospital of Kunming Medical University, Kunming, China; ^2^Key Laboratory of Animal Models and Human Disease Mechanisms of Chinese Academy of Sciences, Kunming Institute of Zoology, Kunming, China; ^3^College of Forensic Medicine, Kunming Medical University, Kunming, China; ^4^Department of Neurosurgery, The Pu’er People’s Hospital, Pu’er, China; ^5^Department of Neurosurgery, Kunming First People’s Hospital, Kunming, China; ^6^Department of Clinical Oncology, Queen Elizabeth Hospital, Hong Kong, Hong Kong SAR, China

**Keywords:** low-grade glioma, prognostic biomarkers, cell proliferation, cell migration, drug sensitivity

## Abstract

**Background:**

Non-SMC condensin I complex subunit G (NCAPG) is expressed in various human cancers, including gliomas. However, its biological function in glioma remains unclear. The present study was designed to determine the biological functions of NCAPG in glioma and to evaluate the association of NCAPG expression with glioma progression.

**Methods:**

Clinical data on patients with glioma were obtained from The Cancer Genome Atlas (TCGA), the Chinese Glioma Genome Atlas (CGGA), the Gene Expression Omnibus (GEO), and the Rembrandt and Gravendeel databases. The correlations among NCAPG expression, pathological characteristics, and clinical outcome were evaluated. In addition, the correlations of NCAPG expression with immune cell infiltration and glioma progression were analyzed.

**Results:**

NCAPG expression was higher in gliomas than in adjacent normal tissues. Higher expression of NCAPG in gliomas correlated with poorer prognosis, unfavorable histological features, absence of mutations in the isocitrate dehydrogenase gene (*IDH*), absence of chromosome 1p and 19q deletions, and responses to chemoradiotherapy. Univariate and multivariate Cox analysis demonstrated, in addition to patient age, tumor grade, absence of *IDH* mutations, and absence of chromosome 1p and 19q deletions, NCAPG expression was independently prognostic of overall survival, disease-free survival, and progression-free survival in patients with glioma. In addition, high expression of NCAPG correlated with tumor infiltration of B cells, CD4+ T cells, CD8+ T cells, neutrophils, macrophages, and dendritic cells. Gene set enrichment analysis (GSEA) indicated that high NCAPG expression was associated with cell proliferation and immune response-related signaling pathways. NCAPG knockdown in glioma cell lines significantly reduced cell survival, proliferation, and migration.

**Conclusion:**

NCAPG expression correlates with glioma progression and immune cell infiltration, suggesting that NCAPG expression may be a useful prognostic biomarker for glioma.

## Introduction

Gliomas are intracranial tumors highly resistant to treatment, with high recurrence and mortality rates (1). Gliomas have been classified into four grades with grades III and grade IV usually leading to poor clinical outcomes (2). Lower-grade gliomas (LGG) can progress to higher-grade gliomas (GBM), which are resistant to chemotherapy (3). Despite various treatment modalities, including surgical intervention, postoperative adjuvant chemoradiotherapy, and immunotherapy, patients with gliomas exhibit poor prognoses (4). Therefore, it is imperative to identify potential prognostic biomarkers and understand the molecular mechanisms regulating glioma progression.

Non-SMC condensin I complex subunit G (NCAPG) is a mitosis-associated chromosomal condensing protein that plays an important role in cancer progression (5). High levels of NCAPG have been associated with poor prognosis in patients with prostate cancer and non-small cell lung cancer (NSCLC) (6, 7). Moreover, NCAPG is widely overexpressed in lung cancer cells, and the induction of NACPG overexpression through activation of the TGF-β signaling pathway was found to promote the progression of lung adenocarcinoma (8). However, the pattern of NCAPG expression, its prognostic value, and its correlation with the tumor microenvironment in glioma remain unclear.

To understand the potential role of NCAPG in glioma, this study investigated the diagnostic and prognostic significance of NCAPG in glioma by data mining of datasets from the Chinese Glioma Genome Atlas (CGGA) and The Cancer Genome Atlas (TCGA). Subsequently, gene ontology (GO) analysis and gene set enrichment analysis (GSEA) were performed to determine the possible biological functions and pathways of NCAPG in glioma. The relationship between NCAPG expression and the infiltration of immune cells in glioma was assessed in the Tumor Immune Estimation Resource (TIMER) database. In addition, the role of NCAPG in glioma expression was analyzed experimentally by immunohistochemistry (IHC), qRT-PCR, and by growth curve, transwell and wound healing assays. These findings indicate that NCAPG can regulate the infiltration of immune cells into gliomas and that the level of NCAPG expression may be a prognostic biomarker in patients with these tumors.

## Materials and Methods

### Analysis of the Expression of NCAPG

The expression of NCAPG in glioma was assessed in several public databases, including the TCGA (3), CGGA (9), GEO (10), and CGGA (9) databases. The prognostic value of NCAPG expression in various cancers was analyzed using the gliovis (11) and GEPIA (12) databases.

### Correlation Between NCAPG Expression and Clinical Features in Glioma

The correlations between NCAPG expression and various clinical characteristics were evaluated using the Xiantaoxueshu database (https://www.xiantao.love/writings). Clinical features evaluated included World Health Organization (WHO) tumor grades, deletion of sequences at chromosomes 1p and 19q, mutations in the gene encoding isocitrate dehydrogenase (IDH), patient age, and responses to radiotherapy and chemotherapy.

### Immune Cell Infiltration Analysis

The associations between NCAPG expression with infiltration into gliomas by B cells, CD4+ T cells, CD8+ T cells, macrophages, neutrophils, and dendritic cells were examined using the TIMER database (13), with analyses performed using the R package GSVA (14).

### KEGG Enrichment Analysis

The potential biological functions of NCAPG in glioma were evaluated using the ClusterProfiler package and GSEA software tools (15, 16)

### Drug Sensitivity Analysis

The relationships between NCAPG expression and sensitivity to drugs were assessed using the Genomics of Drug Sensitivity in Cancer (GDSC) and the Cancer Therapeutics Response Portal (CTRP) databases (17, 18).

### *In Vitro* and siRNA Studies

The glioma cell lines A172, U87, and U251 were purchased from the Kunming Institute of Zoology and were cultured in DMEM medium (Corning) supplemented with 10% fetal bovine serum (FBS) and 1% penicillin/streptomycin at 37°C with 5% CO_2_. The NCAPG siRNAs and a scrambled siRNA for use as a negative control (NC) were synthesized by RiboBio (Guangzhou, China). Cells were transfected with thee siRNAs using Lipofectamine 2000 (Invitrogen) according to the manufacturer’s instructions. Total RNA was obtained 48 h after transfection.

### Quantitative Real-Time PCR

qRT-PCR assays were performed as described (19). In brief, total RNA was extracted from cells and reverse-transcribed using a RT reagent kit (Takara Bio, Beijing, China, Cat# RR047A; TIANGEN Biotech, Beijing, China), as described by their manufacturers. Real-time PCR was performed using FastStart Universal SYBR Green Master Mix (Roche; TIANGEN Biotech, Beijing, China) on an Applied Biosystems 7500 thermal cycler. The primers for NCAPG consisted of 5’-AGTTCTGGCGCTTTCACGAC-3’ (forward) and 5’-GCCCGTCTAACTTCTGGATTTG-3’ (reverse), whereas the primers for the loading control, β-actin, consisted of 5’-CTTCGCGGGCGACGAT-3’ (forward) and 5’-CCATAGGAATCCTTCTGACC-3’ (reverse). The expression of NCAPG mRNA was quantified relative to that of β-actin mRNA using the 2^−ΔΔCt^ method.

### Cell Proliferation Assay

Cell proliferation assays were performed as described (20). Briefly, 1.5x10^4^ cells/well were seed onto 12-well plates, and the numbers of cells were counted daily using a Countstar automatic cell analyzer (Shanghai Ruiyu Biotech Co., China).

### Cell Migration Assay

Cell monolayers in wells of a 6-well plate were scraped in a straight line with a pipette tip. The plates were washed with warm PBS to remove detached cells, and photographed at the indicated time points using a Nikon inverted microscope (Ti-S). Gap widths were calculated with GraphPad Prism 7.0 software. For transwell assays, 1-2×10^4^ cells in 100 µL serum-free medium were plated onto each well of an 8.0-cm, 24-well plate chamber insert (Corning Life Sciences). Medium containing 10% FBS was added to the well below the insert. The plates were incubated for 24 h, and the cells were fixed with 4% paraformaldehyde for 20 min. After washing, the cells were stained with 0.5% crystal violet-blue, and positively stained cells were counted under a light microscope.

### Immunohistochemistry Assay

Immunohistochemistry assays were performed as described previously (20). Briefly, paraffin sections were deparaffinized by xylene, rehydrated with gradient ethanol, and subjected to antigen retrieval. After H_2_O_2_ treatment and blockage with 10% normal goat serum, the slides were incubated with anti-NCAPG antibody (1:200; Proteinch, Shanghai, China), followed by incubation with biotinylated secondary antibody and streptavidin-HRP (Dako, K5007).

### Statistical Analysis

Statistical analyses of the datasets from the TCGA database were performed using R (v.3.6.3) software. The associations between NCAPG and pathologic characteristics were evaluated by Wilcoxon rank sum tests for continuous variables and Chi-square tests for categorical variables. Overall survival (OS), disease-free survival (DFS) and progression-free survival (PFS) were calculated using the Kaplan-Meier method and compared by log-rank tests. Univariate and multivariate Cox regression analyses were performed to assess the correlation between clinical features and OS, DFS, and PFS. For the data regarding the function of NCAPG, statistical analyses were performed using GraphPad Prism 7.0 software. Differences between two groups were assessed using Student’s *t*-tests, and differences among multiple groups were evaluated by one-way ANOVA. *P*–values < 0.05 were considered statistically significant.

## Results

### Correlation Between NCAPG Expression Levels and Clinical and Molecular Characteristics of Glioma Patients

**Figure 1:**
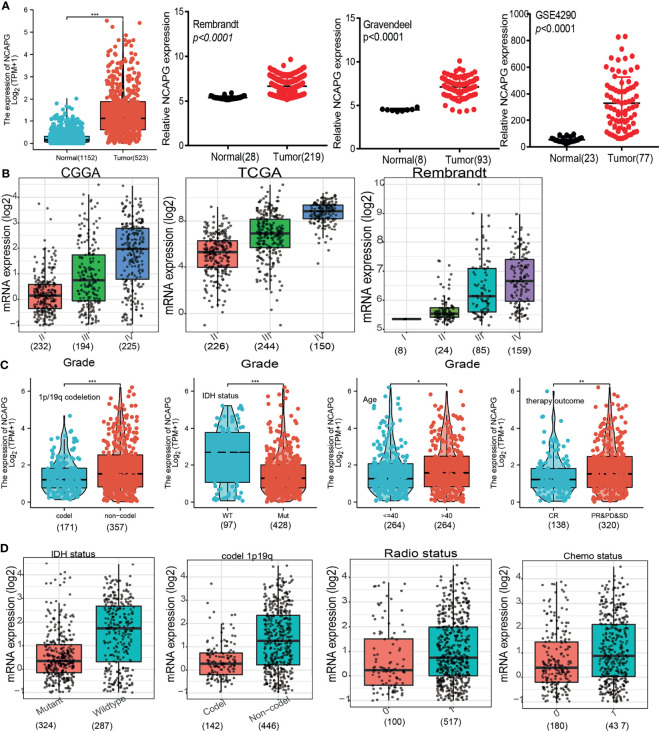
NCAPG expression increases in glioma. **(A–D)** NCAPG expression is significantly up-regulation in glioma examined by the TCGA +GTEx, Rembrandt, Gravendeel, and GEO datasets. **(B)** The expression of NCAPG in various tumor-grade glioma is based on the TCGA, CGGA, and Rembrandt databases. **(C, D)** The correlation between NCAPG expression and different clinical features, including the IDH mutation status, 1p/19q codeletion, age, primary therapy outcome, radiotherapy, and chemotherapy status, Primary therapy outcome: including PD, progressive disease; SD, stable disease. PR, partial response; CR, complete response; The radio status and chemo status 0 representative this patients not received radiation or chemotherapy. The radio status and chemo status 1 representative this patients indeed received radiation or chemotherapy. *P < 0.05, **P < 0.01, ***P < 0.001.

To investigate the association between NCAPG expression and progression of glioma, RNA sequencing data from 1,152 normal brain tissue samples in the GTEx database and from 523 glioma tissue samples in the TCGA database were analyzed. NCAPG expression was significantly higher in glioma tissue than in adjacent normal tissue (P < 0.001); these results were verified by evaluation of datasets from the GEO, Rembrandt, and Gravendeel databases **(****Figure 1A****)**. Evaluation of the correlations between NCAPG expression and the clinical characteristics of glioma patients, as determined by datasets from the CGGA, TCGA, Gravendeel, and Rembrandt databases showed that increased NCAPG expression was associated with higher WHO grade classification **(****Figure 1B****)**. In addition, comparisons of NCAPG expression in gliomas with and without co-deletion of chromosomes 1p and 19q and in gliomas with wild-type and mutant IDH showed that NCAPG expression was higher in gliomas without than with chromosome 1p/19q co-deletions and was significantly higher in IDH-wild type than in IDH mutant gliomas **(****Figures 1C, D****)**. The expression level of NCAPG was also significantly higher in patients aged > 40 years than in those aged ≤ 40 years and was significantly higher in patients who showed partial response (PR), stable disease (SD) or progressive disease (PD) in response to primary treatment than in those who showed complete response (CR) **(****Figure 1C****)**. Moreover, NCAPG expression was significantly higher in glioma patients after than before treatment with radiotherapy and/or chemotherapy **(****Figures 1C, D** and **Table 1****)**.

**Table 1:** The correlation between NCAPG and clinicpathologic characteristic in TCGA-glioma dataset.

Characteristic	Low expression of NCAPG	High expression of NCAPG	P value
n	264	264	
WHO grade, n (%)			< 0.001
G2	158 (33.8%)	66 (14.1%)	
G3	74 (15.8%)	169 (36.2%)	
IDH status, n (%)			< 0.001
WT	27 (5.1%)	70 (13.3%)	
Mut	235 (44.8%)	193 (36.8%)	
1p/19q codeletion, n (%)			0.009
codel	100 (18.9%)	71 (13.4%)	
non-codel	164 (31.1%)	193 (36.6%)	
Age, median (IQR)	38 (31, 49)	43 (33, 55)	0.008

Univariate logistic regression analysis confirmed the association between high NCAPG expression and poor clinicopathological characteristics in glioma patients **(****Table 1****)**. Cox and univariate models showed that NCAPG expression was strongly correlated with WHO grade, primary therapy outcome, IDH status, age, and poorer OS **(****Table 2****)**. Overall, these results demonstrated that increased NCAPG expression correlated significantly with glioma tumorigenesis and progression.

**Table 2:** The relationship between NCAPG clinicopathologic characteristic in the glioma.

Characteristics	Total(N)	Odds Ratio (OR)	P value
WHO grade (G3 vs. G2)	467	5.467 (3.696-8.173)	<0.001
1p/19q codeletion (non-codel vs. codel)	528	1.658 (1.148-2.402)	0.007
Primary therapy outcome (PR&CR vs. PD&SD)	458	0.488 (0.335-0.709)	<0.001
IDH status (Mut vs. WT)	525	0.317 (0.193-0.508)	<0.001
Gender (Male vs. Female)	528	1.183 (0.840-1.669)	0.336
Race (White vs. Black or African American)	509	0.707 (0.287-1.669)	0.433
Age (>40 vs. <=40)	528	1.628 (1.155-2.299)	0.005
Histological type (Oligodendroglioma vs. Astrocytoma)	394	0.799 (0.538-1.187)	0.267
Laterality (Right vs. Left)	517	0.962 (0.681-1.358)	0.825

### Correlation Between NCAPG Expression and Clinical Outcomes in Glioma Patients

**Figure 2:**
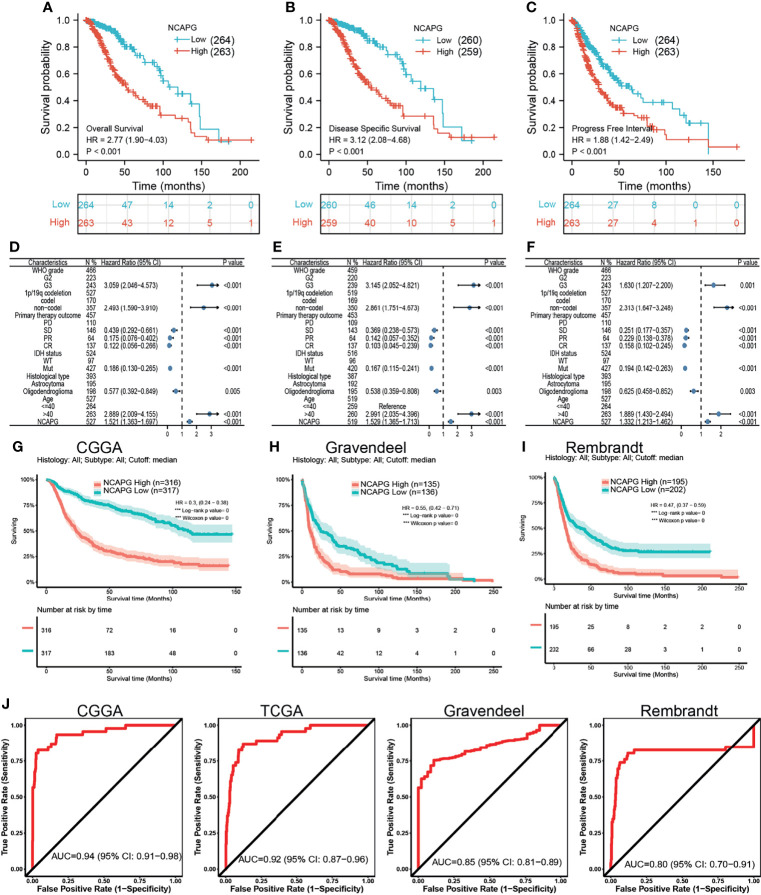
The prognostic value of NCAPG in glioma. **(A–C)** The prognosis of NCAPG in glioma examine by TCGA databases. **(D–F)** The forest plot indicated that the prognosis in the presence of WHO grade, histology type, IDH mutation, 1p/19Q status for **(D)** OS, **(E)** DFS, and **(F)** PFS. **(G–I)** The prognosis of NCAPG in glioma determine by CGGA, Rembrandt, and Gravendeel databases. **(J)** ROC analysis showing the predictive value of NCAPG in glioma based on CGGA, TCGA-glioma, Gravendeel, and Rembrandt databases.

Based on median NCAPG expression in samples from databases, patients were divided into those with high and low levels of NCAPG expression. High NCAPG expression was found to correlate with poorer OS, DFS, and PFS **(****Figures 2A–C****)**. Multivariate analysis, which included WHO grade, co-deletion of chromosomes 1p and 19q, IDH mutation status, and histological type, was utilized to analyze the relationship between NCAPG expression and patient prognosis, including OS, DFS, and PFS **(****Figures 2D–F****)**. These prognostic findings were subsequently validated using datasets from the CGGA, TGGA, Gravendeel, and Rembrandt databases. These analyses confirmed that OS was lower in patients with high than low NCAPG expression **(****Figures 2G–I****)**, indicating that high expression of NCAPG is likely an indicator of poor prognosis in glioma patients. In addition, ROC curve analysis of NCAPG expression of patients in the CGGA, TGGA, Gravendeel, and Rembrandt datasets yielded areas under the curve (AUCs) of 0.94, 0.92, 0.85, and 0.80, respectively **(****Figures 2J****)**. Taken together, these findings suggest that NCAPG is a potential biomarker for prognosis in patients with glioma.

**Figure 3:**
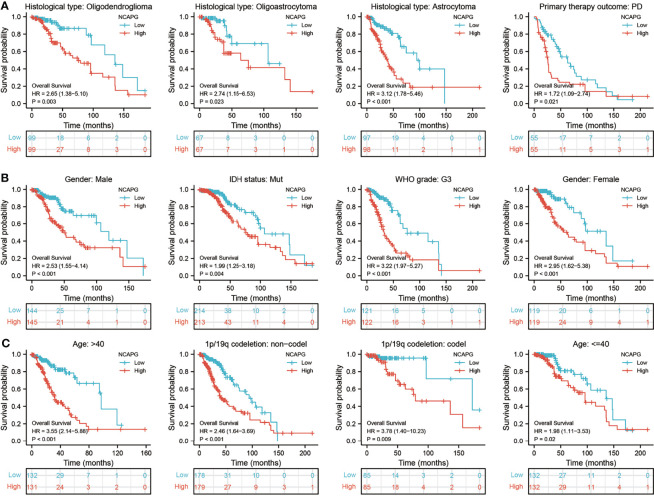
Analysis of the prognostic value of NCAPG in different glioma subgroups. Analysis of the prognostic value of NCAPG in different subgroups, including **(A)** histology type, **(B)** sex, IDH mutation status, WHO grade, and **(C)** age, 1p/19Q status.

Assessment of the prognostic value of NCAPG expression in glioma patients subgrouped by histological type, sex, IDH mutation status, WHO grade, chromosome 1p/19q co-deletion, and age showed that high expression of NCAPG was associated with poor prognosis in all of these groups **(****Figures 3A–C****)**. Univariate analysis showed that NCAPG expression correlated with WHO grade (G3 vs. G2), chromosome 1p/19q co-deletion (no vs. yes), primary therapy outcome (PR and CR vs. PD and SD), IDH status (mutation vs. WT), and age (>40 vs ≤40 years) **(****Table 3****)**. These results suggested that gliomas with high expression of NCAPG were associated with poor outcomes in response to treatment.

**Table 3:** Univariate regression and multivariate survival model of prognostic covariates in patients with glioma.

Characteristics	Total(N)	Univariate analysis		Multivariate analysis	
		Hazard ratio (95% CI)	P value	Hazard ratio (95% CI)	P value
WHO grade	466				
G2	223	Reference			
G3	243	3.059 (2.046-4.573)	<0.001	2.376 (1.267-4.457)	0.007
1p/19q codeletion	527				
codel	170	Reference			
non-codel	357	2.493 (1.590-3.910)	<0.001	1.131 (0.540-2.367)	0.745
Primary therapy outcome	457				
PD	110	Reference			
SD	146	0.439 (0.292-0.661)	<0.001	0.473 (0.256-0.873)	0.017
PR	64	0.175 (0.076-0.402)	<0.001	0.194 (0.059-0.637)	0.007
CR	137	0.122 (0.056-0.266)	<0.001	0.232 (0.097-0.556)	0.001
IDH status	524				
WT	97	Reference			
Mut	427	0.186 (0.130-0.265)	<0.001	0.502 (0.273-0.924)	0.027
Histological type	393				
Astrocytoma	195	Reference			
Oligodendroglioma	198	0.577 (0.392-0.849)	0.005	0.652 (0.355-1.198)	0.168
Age	527				
<=40	264	Reference			
>40	263	2.889 (2.009-4.155)	<0.001	4.111 (2.335-7.236)	<0.001
NCAPG	527	1.521 (1.363-1.697)	<0.001	1.148 (0.950-1.387)	0.152
Gender	527				
Female	238	Reference			
Male	289	1.124 (0.800-1.580)	0.499		
Race	508				
Black or African American	22	Reference			
White	486	0.686 (0.319-1.471)	0.333		

Primary therapy outcome: including PR, CR, SD, and PD. PD, progressive disease; SD, stable disease; PR, partial response; CR, complete response.

**Figure 4:**
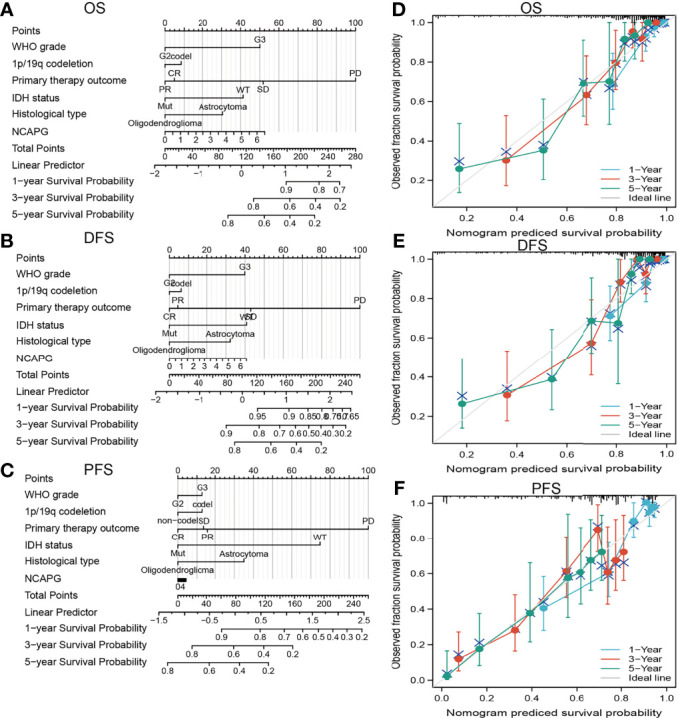
Construction a nomogram to predict the prognosis of NCAPG in glioma. Construction of a nomogram to predict the **(A)** OS, **(B)** DFS, and **(C)** PFS in patients with glioma. The calibration curve used to display the TCGA-glioma cohort for **(D)** OS, **(E)** DFS, and **(F)** PFS.

The ability of a nomogram that included NCAPG expression, histological type, sex, IDH mutant status, WHO grade, chromosome 1p/19q co-deletion, and age to accurately predict prognosis in glioma patients was tested. This nomogram was found to predict 1-, 3-, and 5-year OS, DFS, and PFS in patients with glioma **(****Figures 4A–F****)**.

### Co-Expression Analysis and Gene Ontology Enrichment Analysis

**Figure 5:**
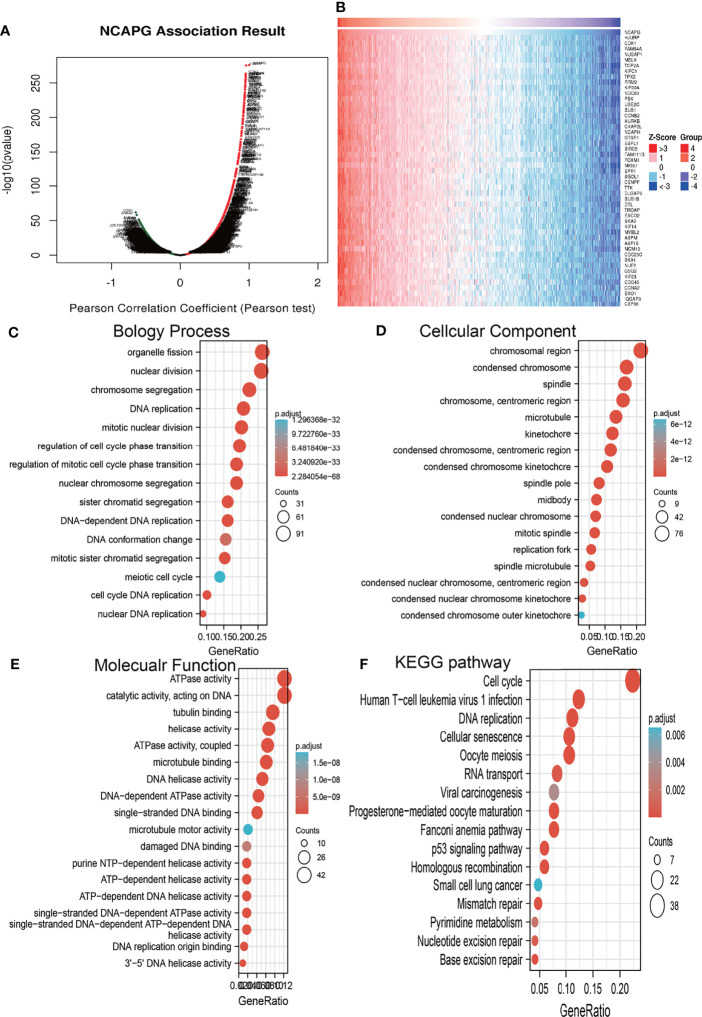
Analysis of the biological function of NCAPG in glioma. **(A, B)** The co-expression gene of NCAPG in glioma explore by Linkedomics. **(C–F)** The GO and KEGG analysis of genes co-expressed with NCAPG in glioma.

The functions of NCAPG in glioma were assessed using LinkedOmics to determine genes co-expressed with NCAPG. This analysis identified 11,002 genes positively correlated with NCAPG and 9,087 genes negatively correlated with NCAPG (**Figure 5A**). A heatmap was constructed showing the 50 most significant genes positively associated with NCAPG expression in glioma **(****Figure 5B****)**. The ClusterProfiler package was used to evaluate the functional enrichment of 100 genes that positively correlated with NCAPG in glioma, with a bubble chart showing the enrichment results using GO and KEGG tools. Annotations of the GO terms suggested that many of the genes co-expressed with NCAPG were involved in biological processes, such as DNA replication, regulation of the cell cycle phase transition, regulation of the mitotic cell cycle phase transition, nuclear DNA replication, DNA conformational changes, and the meiotic cell cycle **(****Figure 5C****)**. Evaluation of cell components associated with genes co-expressed with NCAPG showed that these genes were expressed primarily in chromosomal regions, condensed chromosomes, chromosomes, centromeric regions, kinetochores, centromeric regions, condensed chromosome kinetochores, spindles, microtubules, condensed nuclear chromosome, spindle poles, mitotic spindles, spindle microtubules, replication forks, and midbodies **(****Figure 5D****)**. Annotation of the molecular function of these GO terms suggested that genes co-expressed with NCAPG were mainly involved in catalytic activity, acting on DNA, including DNA helicase, DNA-dependent ATPase, helicase, ATPase, single-stranded DNA-dependent ATP-dependent DNA helicase, single-stranded DNA-dependent ATPase, ATP-dependent DNA helicase, and purine NTP-dependent helicase activities, as well as with binding to single-stranded DNA, tubulin, microtubules, and DNA replication origins **(****Figure 5E****)**.

KEGG pathway enrichment showed that many genes co-expressed with NCAPG were involved in the cell cycle, DNA replication, the Fanconi anemia pathway, oocyte meiosis, cellular senescence, human T cell leukemia virus 1 infection, homologous recombination, mismatch repair, progesterone-mediated oocyte maturation, the p53 signaling pathway, base excision repair, RNA transport, nucleotide excision repair, pyrimidine metabolism, viral carcinogenesis, and small cell lung cancer **(****Figure 5F**).

### GSEA of NCAPG

**Figure 6:**
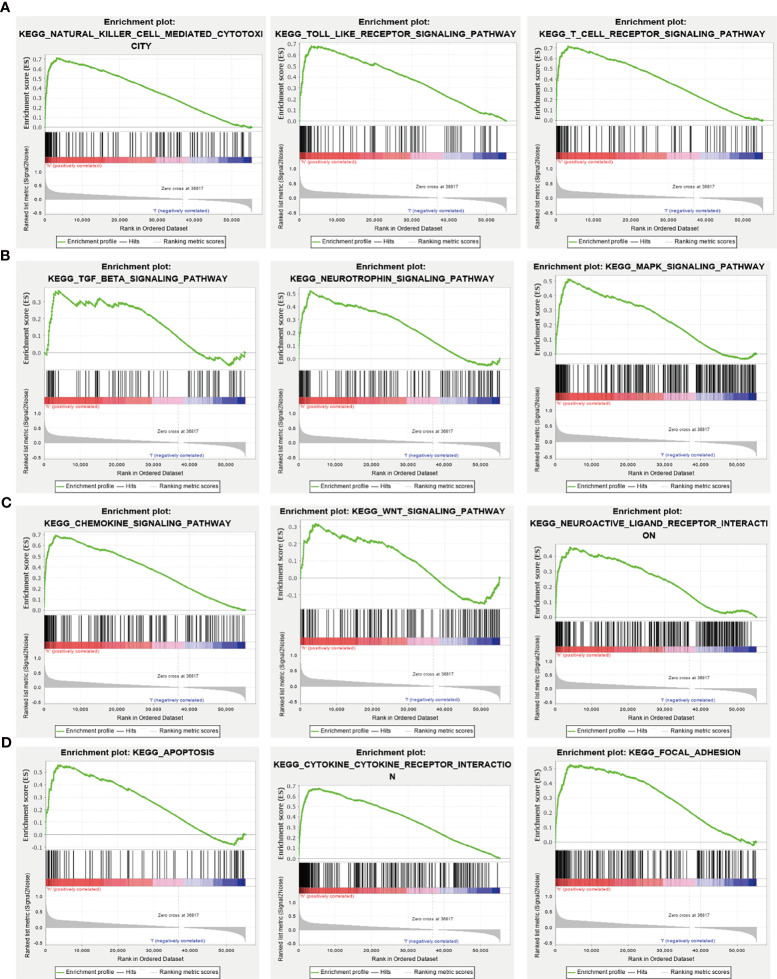
KEGG signaling pathway analysis using GSEA tools. **(A–D)** The involvement of genes coexpressed with NCAPG in glioma signaling pathways as examined by GSEA software.

GSEA tools were utilized to identify the signaling pathways involving NCAPG in glioma (9). High expression of NCAPG was found to be involved in natural killer cell-mediated cytotoxicity; the Toll-like receptor, T cell receptor, TGF-β, neurotrophin, MAPK, chemokine, WNT, and focal adhesion signaling pathways; interactions with neuroactive receptors and cytokine receptors; and apoptosis **(****Figures 6A–D****)**.

### Correlation of NCAPG Expression With Immune Cell Infiltration and Cumulative Survival in Glioma Patients

**Figure 7:**
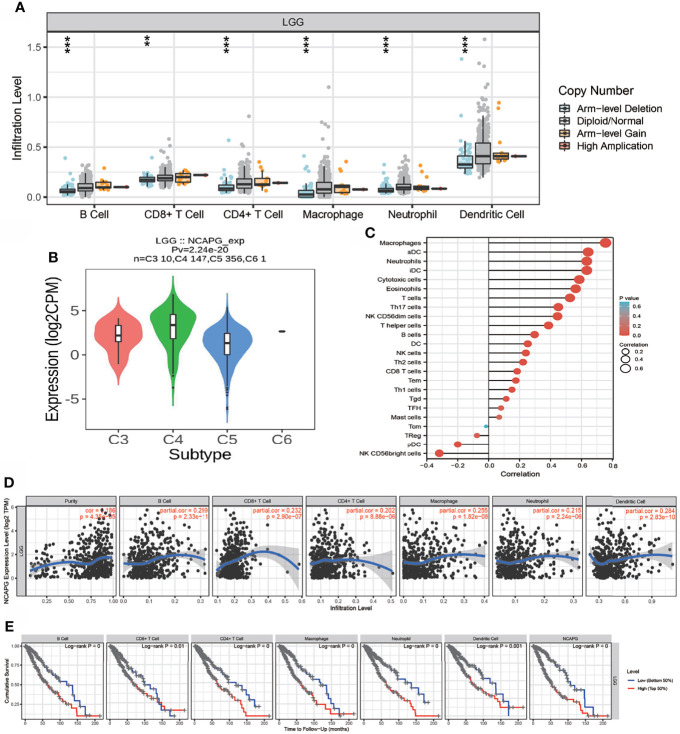
Analysis of the correlation between NCAPG expression and immune cell infiltration. **(A)** The correlation between NCAPG expression and somatic copy number alterations. **(B)** The expression of NCAPG in various immune subtype. **(C, D)** The correlation between NCAPG expression and the infiltration of different immune cells. **(E)** Levels of B cells, CD4+ T cells, CD8+ T cells, dendritic cells, macrophages, and neutrophils are correlated with the cumulative survival rate in glioma. ** p < 0.01, ***p < 0.001.

**Figure 8:**
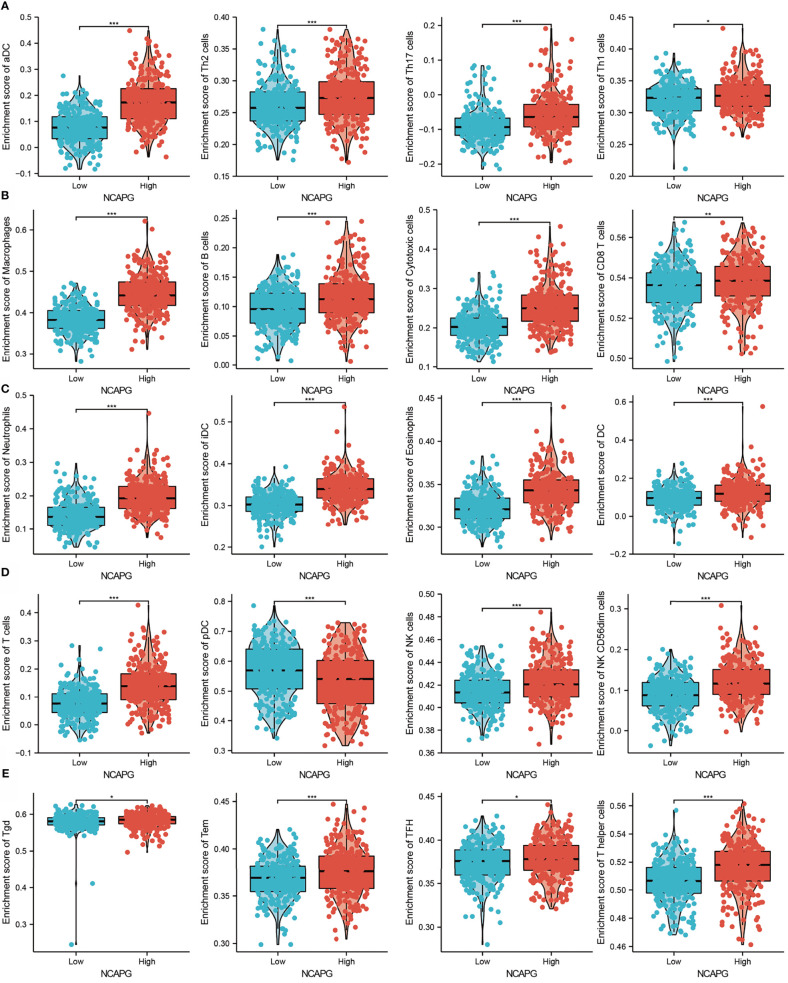
Correlation between NCAPG expression and immune cell infiltration. **(A–E)** Comparison of the levels of infiltration of multiple subtypes of immune cells in the high- and low- expression groups, *P < 0.05, **P < 0.01, ***P < 0.001.

Because GSEA showed that NCAPG expression correlated with the immune response-related signaling pathway, we further examined the correlation between NCAPG expression and immune cell infiltration in glioma tissues. Using TIMER database analysis, we found that alterations in the somatic copy number of NCAPG correlated significantly with infiltration of B cells, CD4+ T cells, CD8+ T cells, neutrophils, macrophages, and dendritic cells into gliomas **(****Figure 7A****)**. Analysis of the expression of NCAPG in immune subtypes of glioma showed that NCAPG was mainly highly expressed in the C4 subtype **(****Figure 7B****)** (21, 22). Moreover, NCAPG expression was positively correlated with immune cell infiltration into gliomas in general and with the infiltration of B cells, CD4+ T cells, CD8+ T cells, macrophages, neutrophils, and dendritic cells in glioma **(****Figures 7C, D****)**. Cox proportional hazard model analysis confirmed that the numbers of B cells, CD8+ T cells, CD4+ T cells, macrophages, neutrophils, and dendritic cells infiltrating into gliomas, as well as NCAPG expression, were associated with a poorer prognosis in glioma patients **(****Figure 7E****)**. Analysis of the effects of high and low levels of NCAPG on immune characteristics of various subtypes of immune cells showed that NCAPG expression significantly affected the levels of infiltration of macrophages, aDCs, neutrophils, iDCs, cytotoxic cells, eosinophils, T cells, Th17 cells, NK CD56dim cells, T helper cells, B cells, DCs, NK cells, Th2 cells, CD8 T cells, Tem cells and Th1 cells **(****Figures 8A–E****)**.

### NCAPG Knockdown Inhibits Glioma Cell Growth and Migration

**Figure 9:**
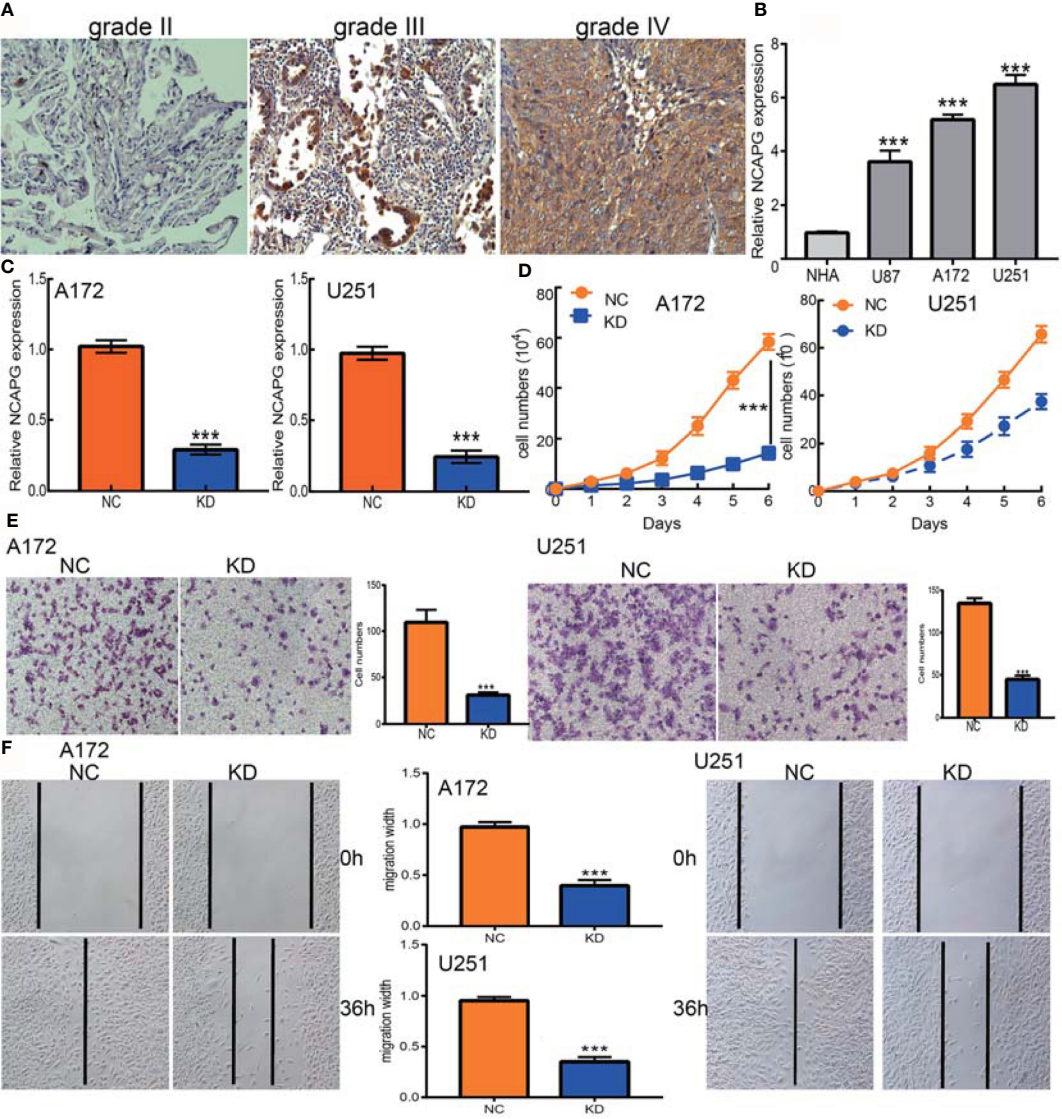
NCAPG depletion inhibits GBM cell proliferation and migration. **(A)** Immunohistochemistry detection of NCAPG in normal brain tissue, glioma, and HGG. **(B)** The expression of NCAPG in normal human astrocytes cells (NHA) and GBM cell lines (U87, A172, and U251). **(C)** The NCAPG knockdown efficiency in A172 and U251 cells verified by qRT-PCR assay. **(D)** NCAPG knockdown significantly inhibited A172 and U251 cell proliferation examined by growth curve assay. **(E, F)** NCAPG knockdown significantly inhibited A172 and U251 cell migration as examined by transwell and wound healing assays. Scale bar =50 μm. NC=Negative control, KD=NCAPG Knockdown, ***P < 0.001.

The association between NCAPG expression and glioma was evaluated immunohistochemically, using antibody to NCAPG to detect the protein level in glioma tissues. The level of expression of NCAPG protein was found to increase with increasing glioma grade **(****Figure 9A****)**. Furthermore, qRT-PCR assays showed that NCAPG was highly expressed in glioma cell lines, especially in A172 and U251 cells, but not in normal human astrocytes **(****Figure 9B****)**. Knockdown of NCAPG in glioma cell lines, as verified by qRT-PCR assay **(****Figure 9C****)**, significantly reduced the proliferation of glioma cells **(****Figure 9D, E****)**. Evaluation of the effects of NCAPG knockdown on the migration ability of glioma cells, as determined by transwell and wound healing assays, showed that knockdown of NCAPG inhibited cell migration **(****Figures 9E, F****)**. Collectively, these results showed that NCAPG was highly expressed in glioma cells and was significantly associated with glioma cell proliferation and migration.

## Discussion

Gliomas are the most common type of malignant tumor of the central nervous system. Patients with glioma generally have poor clinical outcomes, with 5-year survival rate in patients with high-grade glioma being only 10% (23). Identifying potential prognostic biomarkers and understand the molecular mechanisms regulating glioma progression are therefore important. Studies have evaluated the prognostic roles and molecular mechanisms of NCAPG in various tumor types. For example, increased expression of NCAPG has been associated with a poor prognosis in patients with gastric cancer (24). Moreover, NCAPG has been shown to modulate the SRC/STAT3 signaling pathway and confer resistance to trastuzumab in HER2-positive breast cancer cells (25). NCAPG has been reported that to promote hepatocellular carcinoma (HCC) cell proliferation and migration (26) and to be a potential biomarker of endometrial cancer progression and prognosis (27). However, the function of NCAPG in glioma remains incompletely understood.

To our knowledge, the present study is the first to comprehensively evaluate NCAPG expression and its association with clinical and prognostic outcomes in glioma using various public databases, including the CGGA, TCGA, GEO, Rembrandt, and Gravendeel datasets. We found that NCAPG was significantly overexpressed in gliomas and that increased NCAPG expression correlated significantly with poor outcomes, tumor grade, age, IDH mutation status, and chromosome 1p/19q co-deletion status, as well as with the outcomes of radiotherapy and chemotherapy. Cox and univariate analyses showed that NCAPG expression correlated positively with WHO grade, primary therapy outcome, IDH mutation status, age, and poor OS in patients with glioma. Based on multivariate Cox analysis, a nomogram was constructed to predict the prognosis of patients with glioma based on the expression of NCAPG and to stratify glioma patients with better performance. Furthermore, GSEA showed that high NCAPG expression was correlated positively with natural killer cell-mediated cytotoxicity; the Toll-like receptor, T-cell receptor, TGF-β, neurotrophin, MAPK, chemokine, WNT, and focal adhesion signaling pathways; with neuroactive ligand-receptor and cytokine-cytokine receptor interactions; and apoptosis.

Immunotherapy plays an increasingly important role in standard cancer treatment (28), as it can recruit tumor-infiltrating T cells to eradicate tumor cells (29). In glioma, tumor-infiltrating CD4+ T cells play an important role in immune regulation (30). GSEA showed that NCAPG expression was significantly involved in natural killer cell-mediated cytotoxicity and the Toll-like receptor and T-cell receptor signaling pathways. T cell receptor signaling plays a crucial role in immune regulatory processes in glioma (31). Analysis of immune cell infiltration in the present study showed that high NCAPG expression was significantly and positively associated with levels of B cells, CD8+ T cells, CD4+ T cells, macrophages, neutrophils, and dendritic cells in gliomas. Moreover, Cox proportional hazard analysis showed that populations of B cells, CD8+ T cells, CD4+ T cells, macrophages, neutrophils, and dendritic cells, along with NCAPG expression, were significantly associated with poor OS in glioma patients. Taken together, these results suggest that NCAPG might affect immune cell infiltration, making NCAPG expression a predictive biomarker for the effects of immunotherapy in patients with glioma.

Both immunohistochemistry and qRT-PCR assays showed that NACPG was highly expressed in glioma tissue and glioma cell lines. The biological functions of NCAPG in these cells were evaluated by NCAPG knockdown, which significantly inhibited glioma cell proliferation and migration. These results suggest that NCAPG may act as an oncogene in glioma, but additional studies are needed to confirm these findings.

This study had several limitations. Although we explored the correlation between NCAPG and immune cell infiltration in glioma patients, we did not determine the function of NCAPG in regulating the tumor microenvironment in glioma. In addition, we showed that depletion of NCAPG could inhibit the cell migration of glioma cells, the potential molecular mechanisms of NCAPG in cancer metastasis remain unclear. Furthermore, the present study assessed the expression and biological roles of NCAPG in databases of patients with glioma and cultured cells, not *in vivo*. Additional studies are required to assess the function of NCAPG in glioma metastasis and in regulating the glioma tumor microenvironment.

Overall, these results confirmed that NCAPG could serve as a potential novel prognostic biomarker in patients with glioma. Moreover, underlying evidence indicated that NCAPG regulates immune cell infiltration in the glioma tumor microenvironment. These findings can therefore enhance current understanding of not only the role of NCAPG but also its translational use in glioma prognosis and immunotherapy.

## Conclusion

In summary, the findings of this study showed that NCAPG expression was increased in glioma tissues and that its high expression correlated with malignant progression of gliomas. Knockdown of NCAPG in glioma cells reduced their proliferation and migration activities. These results show that NCAPG plays a critical role in glioma progression, and may be useful as a potential novel prognostic biomarker in patients with these tumors.

## Data Availability Statement

The original contributions presented in the study are included in the article/**Supplementary Material**. Further inquiries can be directed to the corresponding authors.

## Author Contributions

XJ, YS, and XC designed this work. HX, BL, and FZ performed related assay and analyzed data. XH, WC, LL, and JP supervised the study and wrote the manuscript. All authors have read and approved the final version of the manuscript.

## Funding

This work supported by the National Nature Science Foundation of China (82160512) and Yunnan Applied Basic Research Projects (2017FE467 and 2018FE001), Kunming Municipal Health Commission Health Research Projects (2020-04-04-113).

## Conflict of Interest

The authors declare that the research was conducted in the absence of any commercial or financial relationships that could be construed as a potential conflict of interest.

## Publisher’s Note

All claims expressed in this article are solely those of the authors and do not necessarily represent those of their affiliated organizations, or those of the publisher, the editors and the reviewers. Any product that may be evaluated in this article, or claim that may be made by its manufacturer, is not guaranteed or endorsed by the publisher.
