# Supplementary material for: NCAPG as a Novel Prognostic Biomarker in Glioma
Source: Front Oncol. 2022 Feb 23;12:831438. doi: 10.3389/fonc.2022.831438 (PMC8906777; doi:10.3389/fonc.2022.831438)
Supplement: Supplementary file 1 [file DataSheet_1.docx]

Supplementary Material


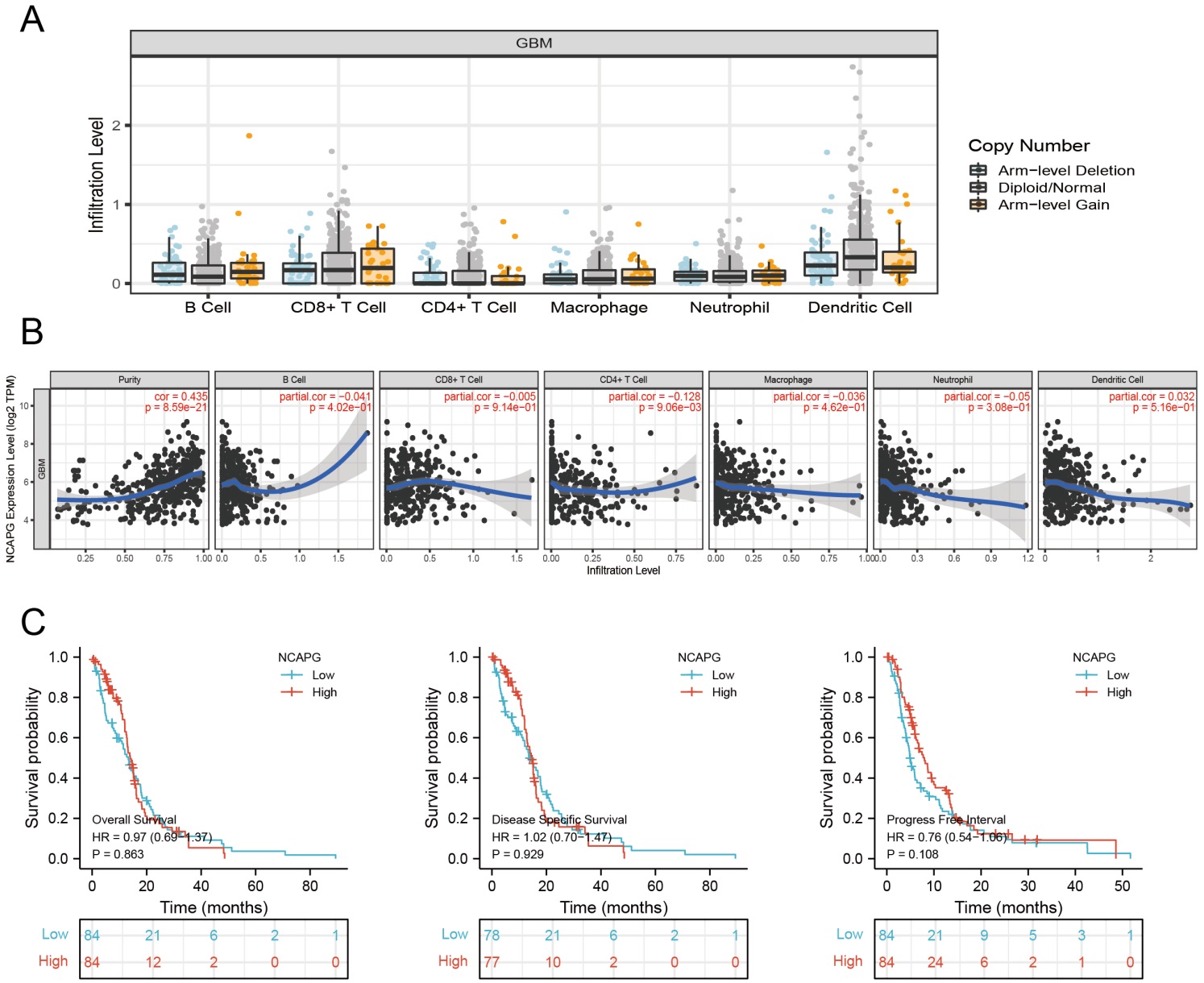


**Supplementary Figure 1. Analysis of the correlation between NCAPG expression and immune cell infiltration in GBM**. (A) The correlation between NCAPG expression and somatic copy number alterations. (B) The correlation between NCAPG expression and the infiltration of different immune cells. (C) Aalysis of overall survival, disease-free survival and progression-free survival in GBM patients.
